# MicroRNA-181a promotes angiogenesis in colorectal cancer by targeting SRCIN1 to promote the SRC/VEGF signaling pathway

**DOI:** 10.1038/s41419-018-0490-4

**Published:** 2018-04-19

**Authors:** Wu Sun, Xiaojun Wang, Jialu Li, Chaoying You, Pan Lu, Huijin Feng, Yan Kong, Haiyang Zhang, Yanqing Liu, Ruihua Jiao, Xi Chen, Yi Ba

**Affiliations:** 1 Tianjin Medical University Cancer Institute and Hospital, National Clinical Research Center for Cancer, Key Laboratory of Cancer Prevention and Therapy, Tianjin’s Clinical Research Center for Cancer, Tianjin, 300060 China; 2 Department of Thoracic Surgery, Nanjing Medical University Affiliated Cancer Hospital, Nanjing, Jiangsu 210009 China; 3 Department of Gastroenterology, Tianjin First Center Hospital, 24 Fukang Road, Tianjin, 300192 China; 4 State Key Laboratory of Pharmaceutical Biotechnology, Jiangsu Engineering Research Center for MicroRNA Biology and Biotechnology, NJU Advanced Institute for Life Sciences (NAILS), School of Life Sciences, Nanjing University, 163 Xianlin Road, Nanjing, Jiangsu 210046 China

## Abstract

Colorectal cancer (CRC) is a very common metastatic tumor with active angiogenesis that requires active angiogenesis. Recently, increased microRNA-181a-5p (miR-181a) expression was found to be significantly associated with liver metastasis and poor outcome in CRC patients. In this study, the role of miR-181a in tumor angiogenesis was further investigated. Capillary tube formation assays were used to demonstrate the ability of miR-181a to promote tumor angiogenesis. Bioinformatics analyses identified SRC kinase signaling inhibitor 1 (SRCIN1) as a potential target of miR-181a. Next, two CRC cell lines (HT29 and SW480) were used to clarify the function of miR-181a through SRCIN1 targeting. In addition, the biological effects of SRCIN1 inhibition by miR-181a were examined *in vitro* by quantitative RT-PCR, western blotting and enzyme-linked immunosorbent assay and *in vivo* by Matrigel plug angiogenesis assays and immunohistochemical staining. In clinical samples, Fluorescence in situ hybridization and immunofluorescence were performed to detect the relation between miR-181a and SRCIN1. In addition, SRCIN1 protein and miR-181a expression levels in CRC tissues were also measured by western blot and quantitative real-time polymerase chain reaction. MiR-181a markedly augmented the capability of CRC cells to advance tube formation in endothelial cells *in vitro*. The Matrigel plug assay showed that miR-181a promoted angiogenesis *in vivo*. In conclusion, miR-181a inhibited SRCIN1, which caused SRC to transform from an inactive status to an active conformation and to trigger vascular endothelial growth factor secretion, leading to increased angiogenesis. MiR-181a dysregulation contributes to angiogenesis in CRC, and downregulation of miR-181a represents a promising, novel strategy to achieve an efficient antiangiogenic response in anti-CRC therapy.

## Introduction

MicroRNAs (miRNAs) are conserved, small (18~22 nucleotides) non-coding RNAs that play important roles in physiological and pathological processes by regulating target gene expression^1^. As a balancing factor between pro- and antiangiogenic progression, miRNA can modulate the appropriate course of events in angiogenesis^2^. In addition to adjusting vessel formation in physiological conditions such as embryonic development and wound healing, emerging evidence has indicated that dysregulated angiogenesis resulting from the aberrant expression of miRNAs plays an important role in tumor progression^3,4^. For example, some miRNAs (miR-23a, miR-21, and the miR-17-92 cluster) promote proangiogenic activity^5–7^, whereas some (miR-29b, miR-29c, and miR-192) inhibit tumor angiogenesis^8–10^. Furthermore, miRNAs represent a potential therapeutic target for the treatment of pathological neovascularization-related diseases because of their influence on multiple pathways^11^.

Colorectal cancer (CRC) is an extremely vascularized tumor that requires angiogenesis to create new blood vessels for further tumor growth and that is characterized by rapid recurrence and poor survival^12^. A previous study showed that microRNA-181a-5p (miR-181a) can promote colorectal tumor growth and liver metastasis via the inhibition of WIF-1^13^. Moreover, a high miR-181a expression level was significantly related to worse survival of CRC patients^14,15^, and the expression level of miR-181a was negatively associated with overall survival (OS) of CRC patients with advanced liver metastases^16^. However, the role of miR-181a in CRC angiogenesis has not yet been fully elucidated.

Endothelial cell (EC) proliferation, invasion, and differentiation play key roles in angiogenesis. As an important angiogenic factor, secreted vascular endothelial growth factor (VEGF) promotes EC proliferation and invasion. Thus, targeting VEGF (e.g., with the standard therapy drug bevacizumab) has become an effective strategy for treating CRC. In fact, as a non-receptor protein tyrosine kinase, a large amount of evidence indicates that SRC regulates angiogenesis via the SRC-STAT3-VEGF pathway. As a SRC-related tumor suppressor factor, SRC kinase signaling inhibitor 1 (SRCIN1) inhibits tumor progression and growth. Furthermore, SRCIN1 regulates the focal adhesion kinase pathway^17^, the epidermal growth factor receptor (EGFR) pathway and the Ras/extracellular signal-regulated kinase pathway by inactivating SRC^18^. Therefore, it is probable that SRCIN1 is involved in regulating CRC angiogenesis.

Here, we demonstrated that miR-181a promoted and SRCIN1 suppressed angiogenesis in CRC. SRCIN1 was identified as a direct target of miR-181a in CRC cells. The inhibition of SRCIN1 by miR-181a activates SRC, which increases VEGF secretion, promoting angiogenesis. Our findings emphasized the importance of miR-181a and SRCIN1 in the regulation of angiogenesis and identified miR-181a as a potential therapeutic target for CRC.

## Results

### miR-181a promotes angiogenesis *in vitro*

**Fig. 1:**
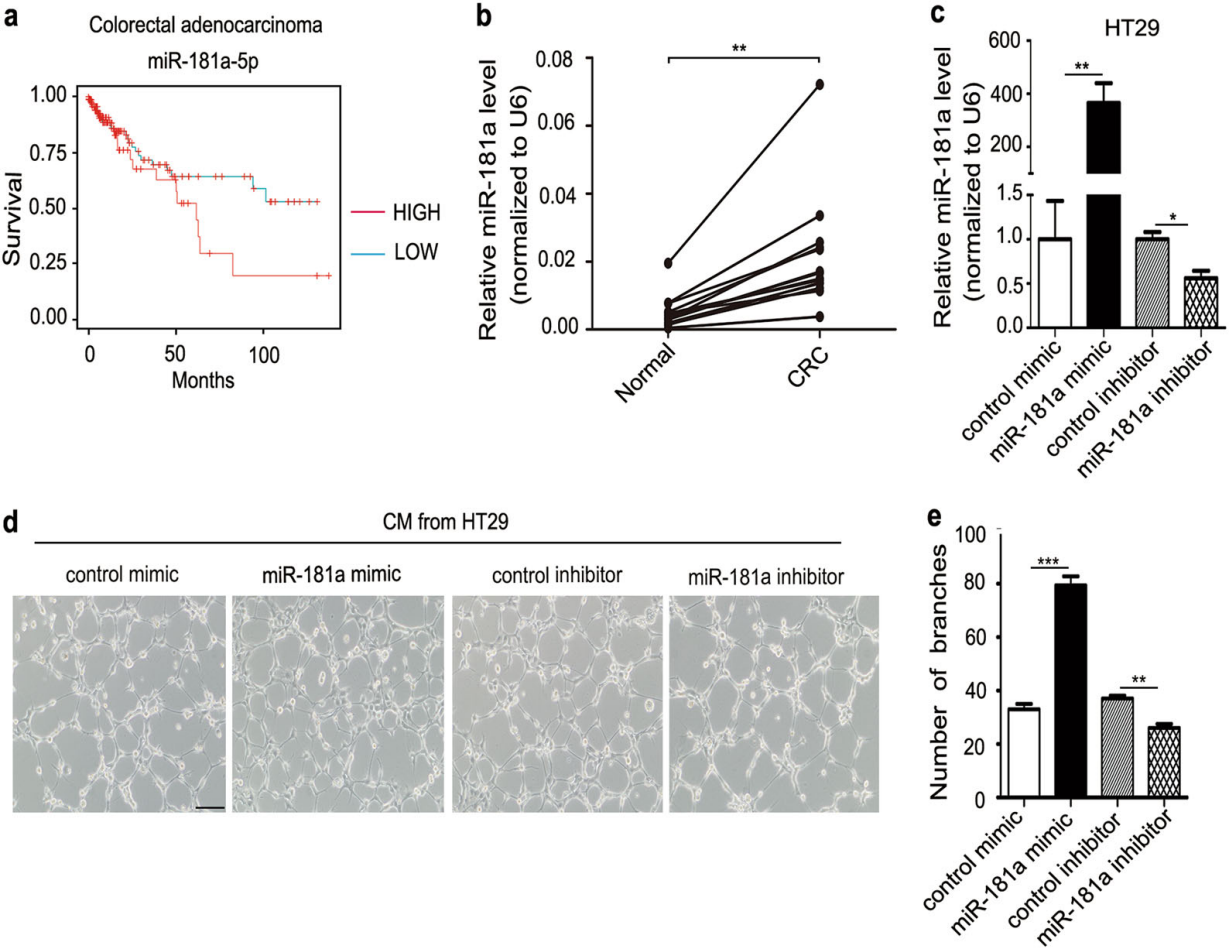
miR-181a promotes angiogenesis *in vitro*. **a** Increased miR-181a expression is associated with a worse 5-year survival in colorectal adenocarcinoma patients. **b** miR-181a is upregulated in tumor tissues compared with adjacent non-tumorous tissues (*n* = 14). **c** Quantitative RT-PCR analysis of miR-181a expression levels in HT29 cells transfected with control mimic, miR-181a mimic, control inhibitor, or miR-181a inhibitor. **d**, **e** Overexpression of miR-181a in HT29 cells promoted tube formation in HUVECs. HUVECs were cultured in the presence of 75% TCM from HT29 cells transfected with control mimic, miR-181a mimic, control inhibitor, or miR-181a inhibitor, scale bars: 100 μm; **d** representative images of tube formation; **e**HUVEC branch number. Data are shown as the mean ± SD of three replicates.*P < 0.05; **P < 0.01; ***P < 0.001

To identify candidate regulators of angiogenesis, we first performed miRNA microarray analysis to screen mature miRNA expression in CRC and normal adjacent tissue (NAT) samples. As is shown in Supplementary Table 1, a panel of miRNAs was found to be dysregulated in CRC; 39 miRNAs were significantly upregulated (fold change > 2) and 48 miRNAs were clearly downregulated (fold change < 0.5) in CRC group. When combined with our previous report^19^, we found that only four miRNAs (miR-199a, miR-181a, miR-199b, and miR-27a) were commonly increased (Supplementary Fig. 1A). Through biological pathway analysis, we found miR-181a were involved in angiogenesis related signaling pathway, like VEGF and VEGFR signaling network (Supplementary Fig. 1B). To further investigate the clinical significance of miR-181a in colorectal carcinoma, we used an online database, YM500^16^, to analyze the OS rate of colorectal adenocarcinoma patients depending on the miR-181a expression level and observed that patients with higher miR-181a expression had worse survival (Fig. 1a). Furthermore, analysis of miRNA expression between eight normal solid tissues and 457 colorectal adenocarcinoma tissues showed that miR-181a was upregulated ~ 13.58-fold in cancer tissues (Supplementary Table 2). To confirm this result, we measured miR-181a expression levels in 14 pairs of CRC tissues and in corresponding normal adjacent tissues and found that miR-181a expression levels were consistently higher in CRC tissues (Fig. 1b).

Angiogenesis is closely related to tumorigenesis and patient survival. To explore the potential biological function of miR-181a in tumor angiogenesis, an *in vitro* capillary tube formation assay was performed by using the HT29 cell line. First, we successfully overexpressed or silenced miR-181a expression in HT29 cells by using a miR-181a mimic or inhibitor, respectively (Fig. 1c). Then, we collected tumor cell-conditioned medium (TCM) from HT29 cells and added it to the culture medium of human umbilical vein endothelial cells (HUVECs). In the presence of TCM from the miR-181a overexpression group, HUVECs developed more capillary-like structures than in the presence of control cell-conditioned medium (Fig. 1d, e). However, TCM from the miR-181a-inhibited HT29 cells suppressed the formation of capillaries (Fig. 1d, e). On the other hand, we determined the function of miR-181a in another CRC cell line, SW480. We successfully overexpressed or silenced miR-181a expression in SW480 cells by using a miR-181a mimic or inhibitor, respectively (Supplementary Fig. 2A). MiR-181a overexpression significantly promoted HUVEC cell tube formation, whereas miR-181a inhibition suppressed tube formation (Supplementary Fig. 2B, C). These data suggested that miR-181a can promote CRC angiogenesis *in vitro*.

### miR-181a promotes angiogenesis *in vivo*

**Fig. 2:**
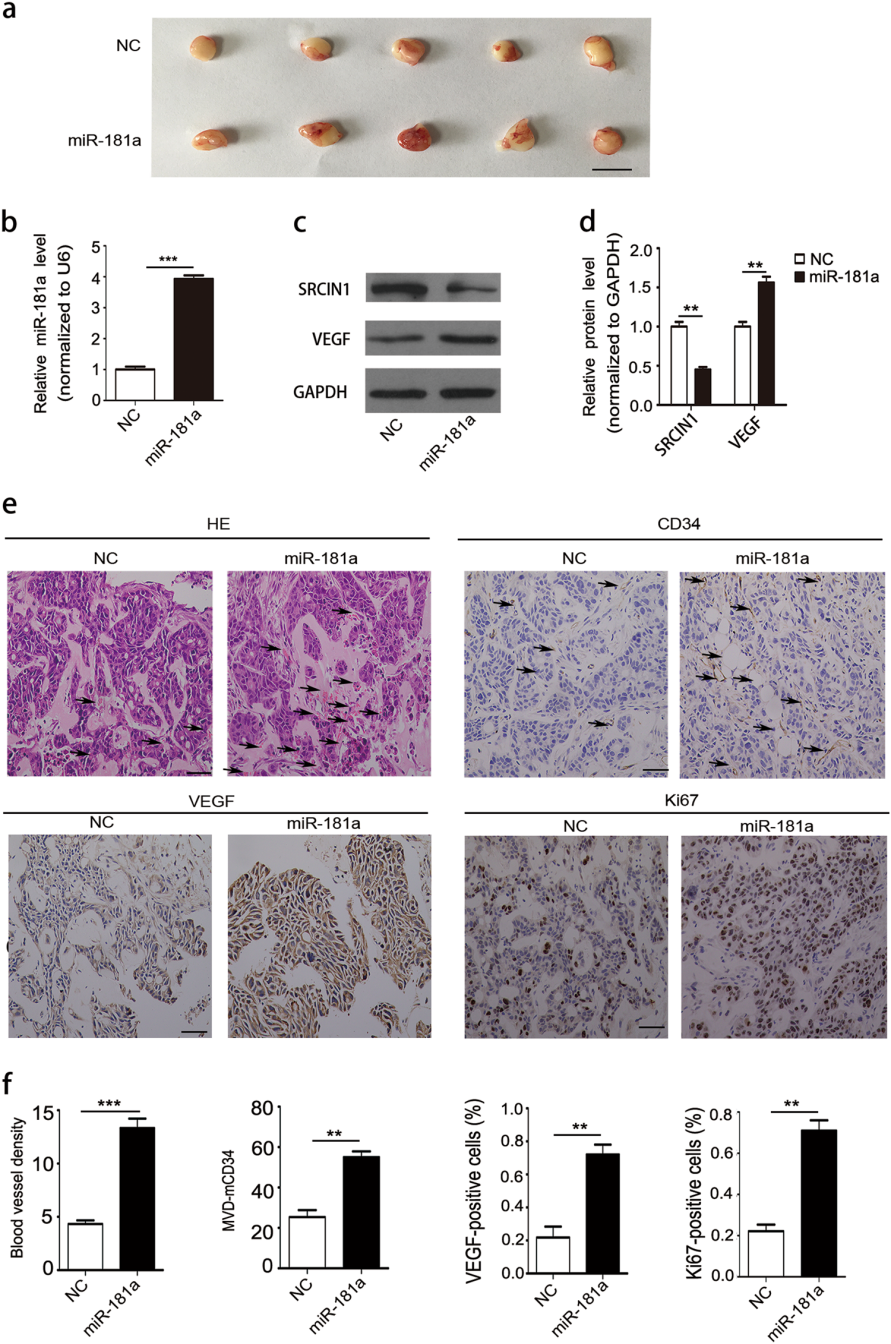
miR-181a promotes angiogenesis *in vivo*. HT29 cells were infected with a control lentivirus or a miR-181a-overexpressing lentivirus. **a** Representative images of tumors from the implanted mice (*n* = 5). **b** Quantitative RT-PCR analysis of miR-181a expression levels in tumors from implanted mice. **c**, **d** Western blotting analysis of SRCIN1 and VEGF protein levels in tumors the implanted mice; **c**representative images; **d** quantitative analysis. **e**, **f** Tumors from implanted mice were subjected to H&E-stained and immunohistochemical staining for CD34, VEGF and Ki-67,scale bars: 50 μm; **e** representative images **f** quantitative analysis. **P < 0.01; ***P < 0.001

We next investigated the effect of miR-181a on CRC angiogenesis *in vivo*. First, we constructed miR-181a-overexpressing HT29 cells by using lentiviral vectors, and the efficiency of lentiviral infection and the level of miR-181a overexpression were determined (Supplementary Fig. 3A, B). Then, the Matrigel plug assay was used to confirm the proangiogenic effect of miR-181a. The plugs were removed 7 days after implantation (Fig. 2a). Subsequently, total RNA and protein were extracted from the plugs and used to evaluate the expression levels of miR-181a, SRCIN1 and VEGF. Tumors from the miR-181a overexpression group showed a significant increase in the expression of miR-181a (Fig. 2b), lower levels of SRCIN1 and higher levels of VEGF compared with control group (Fig. 2c, d). The H&E results showed more newly formed blood vessels in the stained sections from miR-181a-overexpressing plugs than in those from the controls (Fig. 2e, f and Supplementary Fig. 4). Importantly, CD34 staining showed that there were more infiltrating blood vessels in the miR-181a-overexpression group (Fig. 2e, f) than in the control group, suggesting that angiogenesis might be enhanced by miR-181a *in vivo*. Iimmunohistochemical (IHC) results also revealed that VEGF expression was upregulated in miR-181a-overexpressing xenografts (Fig. 2e, f), implying that miR-181a promoted CRC angiogenesis via a mechanism that increases VEGF expression. Moreover, Ki-67 staining indicated that miR-181a could promote HT29 proliferation (Fig. 2e, f).

### Identification of SRCIN1 as a direct target gene of miR-181a

**Fig. 3:**
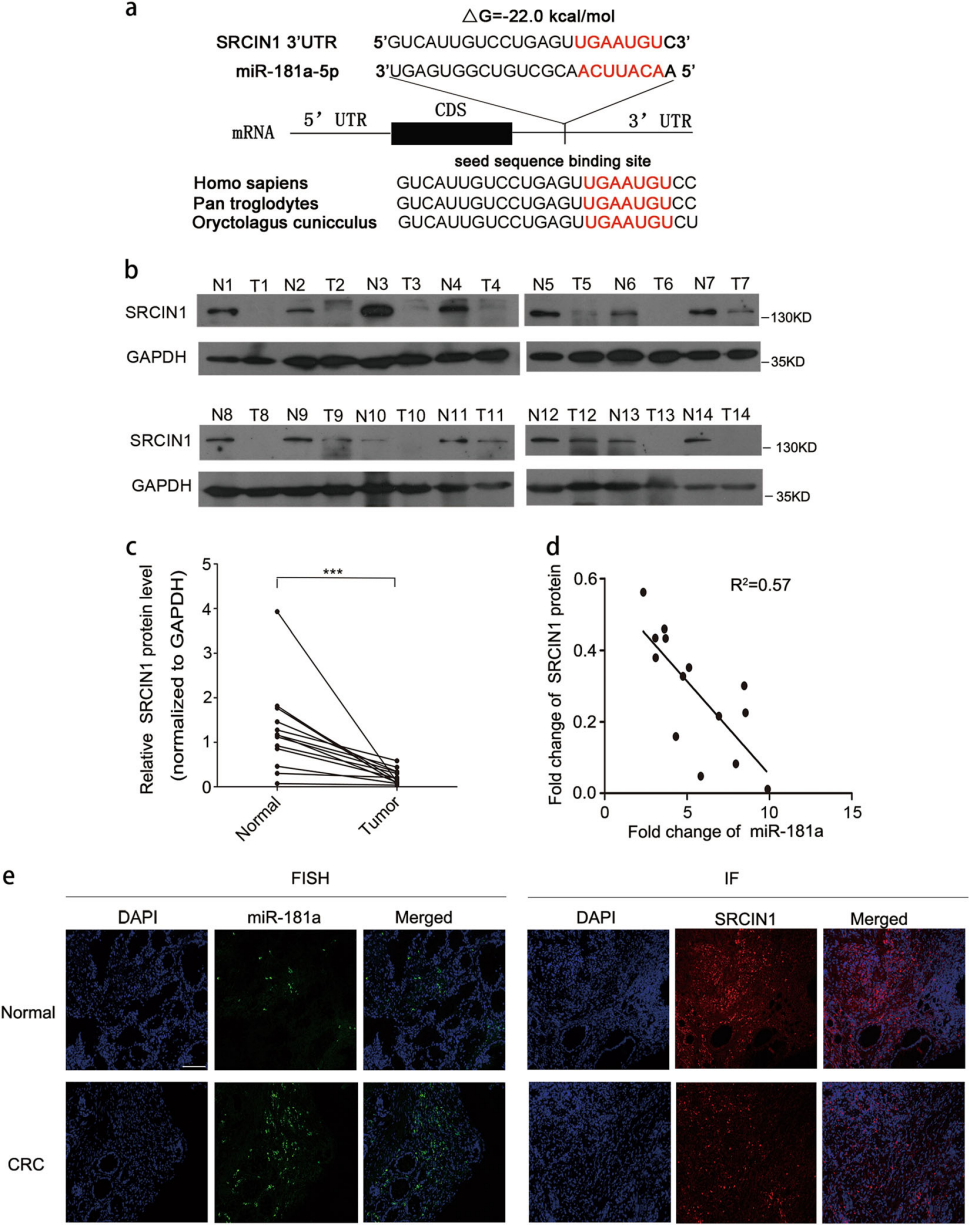
Identification of SRCIN1 as a direct target gene of miR-181a. **a** Sequence pairing between mature miR-181a and the human SRCIN1 3′-UTR. All nucleotides of the seed sequence of the binding site are conserved in several species, including Homo sapiens, Pan troglodytes, and Oryctolagus cuniculus. The predicted free energy values of the hybrids are indicated. **b**, **c** Western blot analysis of SRCIN1 protein expression levels in the same paired CRC and normal tissue samples (*n* = 14); **b** representative images; **c** quantitative analysis. **d** Pearson’s correlation scatter plot of the fold changes in miR-181a and SRCIN1 protein expression in human CRC tissue pairs. **e** Clinical specimens of CRC and normal tissue samples were stained for miR-181a, SRCIN1. Representative images from a tissue microarray are shown, scale bars: 100 μm. ***P < 0.001

To explore the molecular mechanisms responsible for the proangiogenic function of miR-181a, we searched for the downstream target genes of miR-181a by using miRNA target prediction software (TargetScan, PicTar, and RNAhybrid)^20–22^, SRCIN1 was chosen for further experimental confirmation because it was not only predicted to be targeted by miR-181a but also exhibited a potential role in inhibiting SRC/VEGF signaling. According to the software prediction, miR-181a is partially complementary to the 3′-untranslated region (3′-UTR) of human SRCIN1. The minimum free energy value of this hybridization is −22 kcal/mol, indicating a tight interaction. Furthermore, this binding is highly conserved across species (Fig. 3a).

We next investigated whether miR-181a expression levels were inversely correlated with SRCIN1 expression in CRC tissues. First, we used an online database, YM500V3, to perform a meta-analysis of the SRCIN1 expression pattern. The data showed that SRCIN1 expression in the tumor tissues was significantly downregulated compared to that in normal solid tissues (Supplementary Table 2). Furthermore, by measuring SRCIN1 expression levels in the same 14 pairs of CRC tissues and corresponding normal adjacent tissues, we found that SRCIN1 protein expression levels were significantly decreased in CRC tissues compared with those in normal adjacent tissues (Fig. 3b, c). Furthermore, we illustrated the inverse correlation between miR-181a and SRCIN1 protein expression levels by using Pearson’s correlation scatter plots. As shown in Fig. 3d, miR-181a expression was highly inversely correlated with SRCIN1 expression (*R*^2^=0.57). To confirm the relation between miR-181a and SRCIN1, *in situ* hybridization revealed that miR-181a was higher expression and IF showed that SRCIN1 was downregulated in CRC compared with normal tissues (Fig. 3e).Thus, we speculated that the upregulation of miR-181a expression may be responsible for the decreased expression level of SRCIN1 in human CRC tissues, which promotes angiogenesis in CRC.

### miR-181a directly regulates SRCIN1 expression and promotes the SRC/VEGF signaling pathway

**Fig. 4:**
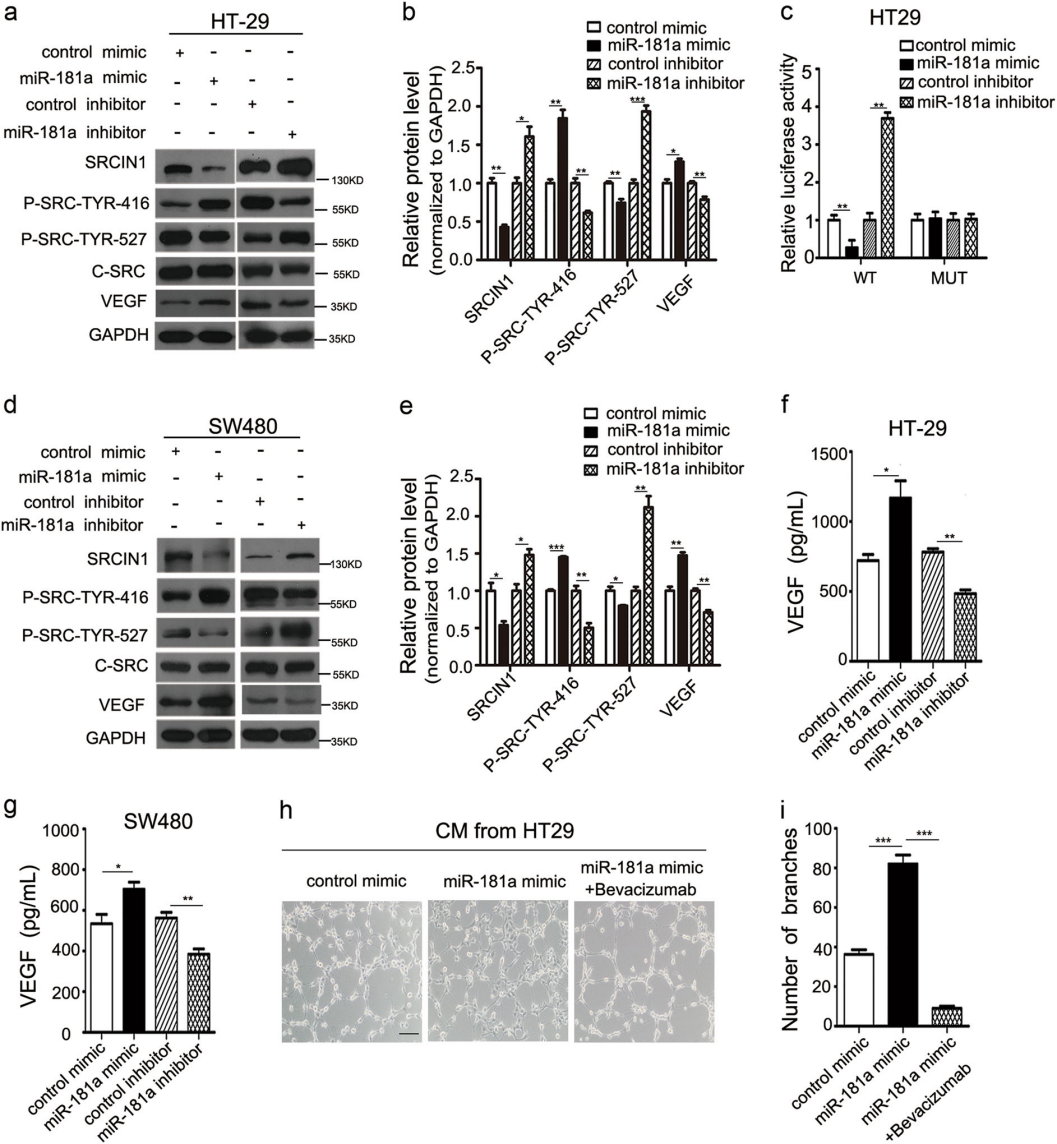
miR-181a directly regulates SRCIN1 expression and promotes the SRC/VEGF signaling pathway. **a**,** b** Western blotting analysis of the protein expression levels of SRCIN1, phosphorylated-SRC (Tyr527), phosphorylated-SRC (Tyr416), c-SRC, and VEGF in HT29 cells after transfection with control mimic, miR-181a mimic, control inhibitor, or miR-181a inhibitor; **a** representative images; **b** quantitative analysis. **c** The relative luciferase activity of HT29 cells transfected with the wild-type or mutant SRCIN1 3′-UTR. **d**, **e** Western blotting analysis of protein expression levels in another cell line (SW480); **d** representative images; **e** quantitative analysis. **f** ELISA analysis of VEGF secretion from HT29 cells after transfection. **g** VEGF secretion detected in TCM from SW480. **h**, **i** HUVECs were cultured in the presence of 75% TCM from HT29 cells transfected with control mimic or miR-181a mimic with or without 0.25 mg/ml bevacizumab, scale bars: 100 μm; **h** representative images of tube formation; **i** HUVEC branch number. Data are shown as the mean ± SD of three replicates.*P < 0.05; **P < 0.01; ***P < 0.001

We manipulated miR-181a expression levels in CRC cell lines to investigate whether this alteration could inhibit SRCIN1 expression. As expected, SRCIN1 protein expression levels dramatically decreased upon miR-181a overexpression, whereas treatment with the miR-181a inhibitor increased SRCIN1 protein expression levels in the HT29 cell line (Fig. 4a, b). However, the alteration of miR-181a had little effect on the SRCIN1 mRNA expression level (Supplementary Fig. 5A). Luciferase reporter analyses showed that miR-181a significantly inhibited the activity of firefly luciferase when co-expressed with the wild-type (WT), but not the mutant, 3′-UTR of SRCIN1 (Fig. 4c), indicating that miR-181a may suppress SRCIN1 expression through its binding at the 3′-UTR of SRCIN1.

The SRC-STAT3-VEGF pathway is vital for CRC angiogenesis, and SRCIN1 is a significant negative regulator of SRC. We investigated whether miR-181a can influence the SRC-VEGF pathway to regulate CRC angiogenesis by suppressing SRCIN1 expression. First, overexpression of miR-181a in HT29 cells downregulated SRCIN1 protein expression and resulted in less phosphorylation of the inhibitory Tyr527 on SRC, which led to an increase in SRC kinase activity, represented by an additional phosphorylation at Tyr416 (Fig. 4a, b). Moreover, the level of VEGF was also upregulated (Fig. 4a, b). In contrast, knocking down miR-181a increased the level of SRCIN1 protein, thus leading to a reduction in SRC activity and the level of VEGF (Fig. 4a, b). Furthermore, we examined the level of VEGF mRNA. As anticipated, VEGF mRNA levels markedly increased upon miR-181a overexpression, whereas treatment with the miR-181 inhibitor decreased VEGF mRNA levels (Supplementary Fig. 5B). Moreover, we repeated the above experiments in another CRC cell line (SW480) to validate the robustness of the test. As expected, miR-181a overexpression also repressed SRCIN1 expression and increased SRC activity and the level of VEGF protein and mRNA in SW480 cells, whereas miR-181a suppression caused the opposite effect (Fig. 4d, e and Supplementary Fig. 5C, D).

Next, using enzyme-linked immunosorbent assay (ELISA) assays, we investigated the effect of the miR-181a-SRCIN1 regulatory axis on VEGF secretion. A significant increase in the secretion of the angiogenic factor VEGF was confirmed in the supernatants of miR-181a-overexpressing HT29 cells (Fig. 4f). Furthermore, ELISA assays in SW480 cells revealed the same trend as seen in HT29 cells (Fig. 4g). We further evaluated whether VEGF is the key proangiogenic factor that promotes tube formation. Bevacizumab, a common anti-VEGF antibody, was used to block VEGF activation^23^. The addition of bevacizumab completely suppressed miR-181a–induced HUVEC tube formation (Fig. 4h, i). These results indicated that miR-181a inhibited SRCIN1 expression and promoted SRC/VEGF signaling.

### miR-181a promotes angiogenesis and SRC/VEGF signaling by its inhibition of SRCIN1

**Fig. 5:**
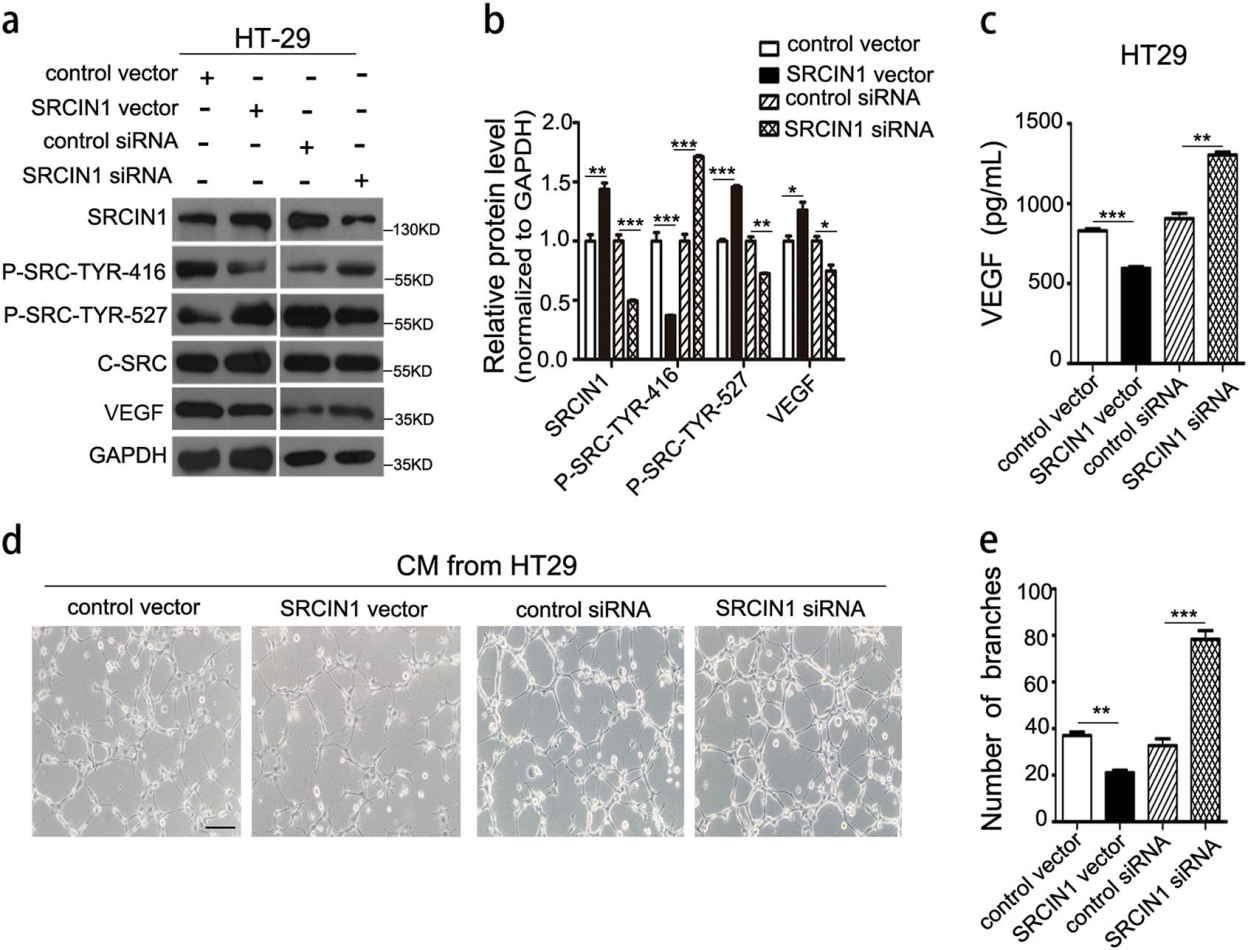
SRCIN1 inhibits angiogenesis and SRC/VEGF signaling. **a**, **b**Western blotting analysis of the protein expression levels of SRCIN1, phosphorylated-SRC (Tyr416), phosphorylated-SRC (Tyr527), c-SRC, and VEGF in HT29 cells transfected with control plasmid, SRCIN1 overexpression plasmid, control siRNA, or SRCIN1 siRNA; **a** representative images; **b** quantitative analysis. **c** ELISA analysis of VEGF secretion from HT29 cells after transfection as described in **a**. **d**,** e** TCM derived from HT29 cells transfected with control plasmid, SRCIN1 overexpression plasmid, control siRNA, or SRCIN1 siRNA were added to HUVECs at a final concentration of 75% TCM, scale bars: 100 μm; **d** representative images of tube formation; **e** HUVEC branch number. Data are shown as the mean ± SD of three replicates. *P < 0.05; **P < 0.01; ***P < 0.001

**Fig. 6:**
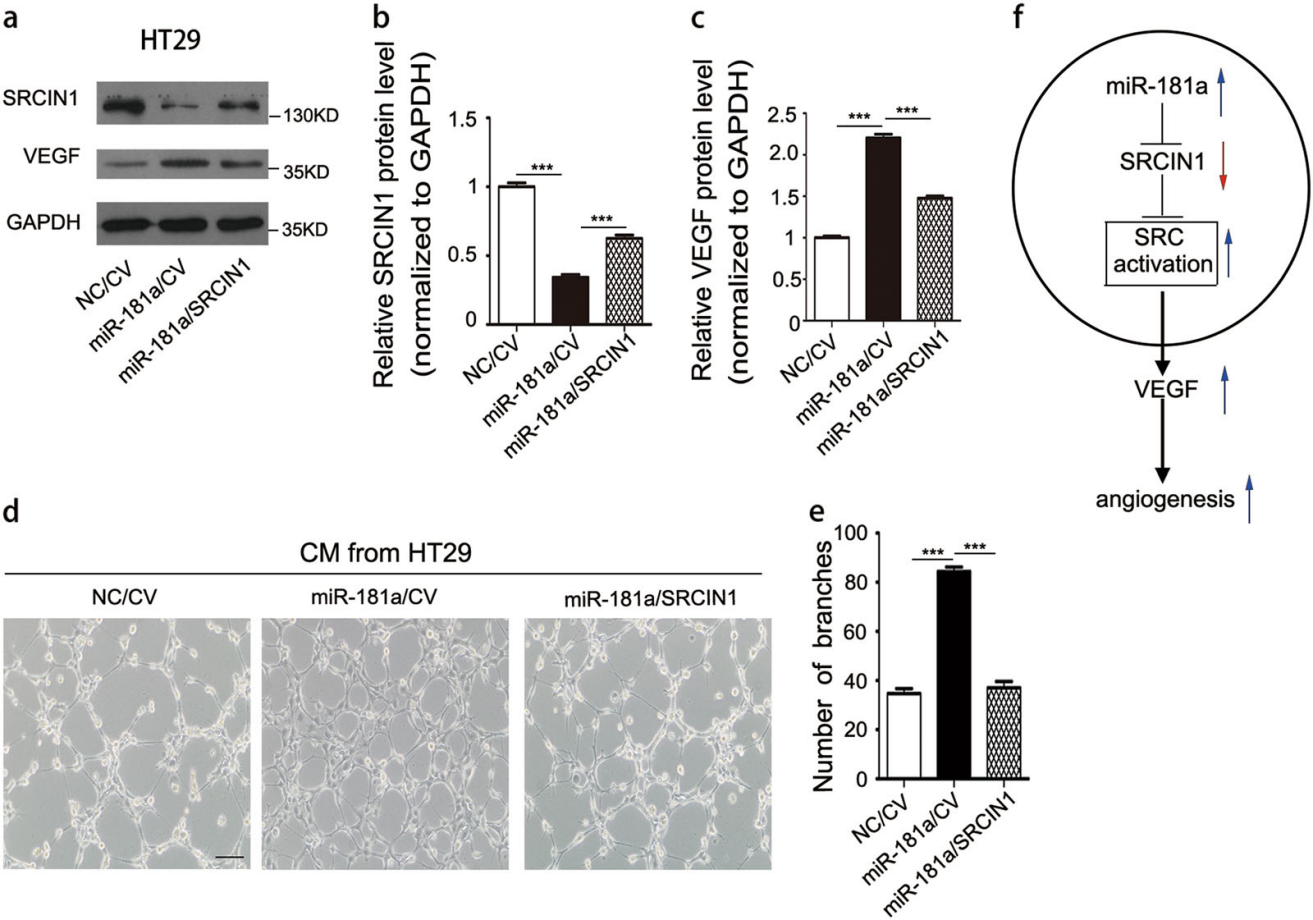
miR-181a promotes angiogenesis and SRC/VEGF signaling by its inhibition of SRCIN1. **a**–**c** Western blotting analysis of protein expression levels in HT29 transfected with control mimic, miR-181a mimic, or miR-181a mimic plus SRCIN1 plasmid;** a** representative images; **b**, **c** quantitative analysis. **d**,** e** TCM derived from HT29 cells cotransfected with NC/CV (lane 1), miR-181a/CV (lane 2), or miR-181a/SRCIN1 (lane 3) were added to HUVECs, scale bars: 100 μm; **d** Representative images of tube formation. **e** HUVEC branch number. **f** Working model of the miR-181a–SRCIN1–SRC–VEGF axis in CRC. Data are shown as the mean ± SD of three replicates. **P < 0.01; ***P < 0.001

To explore whether the angiogenesis-promoting effect of miR-181a was due to its ability to inhibit SRCIN1, we first investigated whether SRCIN1 mediated angiogenesis. We knocked down and overexpressed SRCIN1 in HT29 cells (Fig. 5a, b and Supplementary Fig. 6a). SRCIN1 knockdown significantly activated SRC expression and increased the level of VEGF protein and mRNA, whereas SRCIN1 overexpression caused the opposite effect (Fig. 5a, b and Supplementary Fig. 6B). Furthermore, ELISA assays showed that TCM obtained from SRCIN1 overexpression cells displayed a significant decrease in the level of VEGF compared with TCM obtained from control cells, whereas the downregulation of SRCIN1 promoted VEGF secretion (Fig. 5c). Next, SRCIN1 overexpression significantly disrupted HUVEC cell tube formation. In addition, SRCIN1 depletion promoted HUVEC cell tube formation (Fig. 5d, e). These results showed that SRCIN1 has a vital role in promoting angiogenesis. We then transfected HT29 cells with the overexpression plasmid of SRCIN1 to reverse the effect of miR-181a mimic. As expected, SRCIN1 protein expression was rescued by cotreatment with the overexpression plasmid of SRCIN1 compared with treatment with miR-181a mimic alone (Fig. 6a–c). In contrast, increased VEGF protein expression caused by the miR-181a mimic was reduced by the co-added SRCIN1 overexpression plasmid (Fig. 6a–c). Furthermore, the reintroduction of SRCIN1 into miR-181a-overexpressing HT29 cells exhibited reduced tube formation (Fig. 6d, e), implying that the promoting effect of miR-181a on angiogenesis was mediated by its inhibition of SRCIN1.

## Discussion

Tumors require angiogenesis to provide oxygen and nutrients for unlimited expansion and metastatic dissemination^24,25^. A deeper mechanistic understanding of tumor angiogenesis regulation will contribute to improving therapeutic strategies. Numerous reports have identified miRNAs (e.g. miR-17, miR-92, and miR-21) that display proangiogenic activity^6,7^, but this activity is restricted to ECs. However, as has been widely reported, tumor cells are key initiators and promoters of angiogenesis. Therefore, the angiogenic network will be improved by the identification of imbalances in miRNA regulation in tumor cells and by clarification of the communication occurring between cancer cells and neighboring endothelial cells.

In the present study, we showed that miR-181a is capable of promoting angiogenesis by directly suppressing SRCIN1 expression and promoting the SRC/VEGF pathway, leading to elevated VEGF secretion. Our study demonstrated a vital intercellular communication between tumor cells and endothelial cells, which ultimately leads to angiogenesis. As a famous “oncomiR”, miR-181a has been confirmed to play various roles in tumorigenesis in many kinds of cancers, including ovarian cancer, gastric cancer and breast cancer^26–28^. More importantly, miR-181a could promote chondrosarcoma growth through increasing VEGF expression and secretion^29^. In colorectal carcinoma development, miR-181a has been reported to promote tumor growth and liver metastasis, to be upregulated by oncogenic KRAS^30^ and to promote cell viability^13^. In clinical studies, the miR-181a expression level is not only related to poor survival but also predicts short PFS in CRC patients receiving EGFR-targeted therapy^14,15^. What is more, when patients with mCRC given the first-line treatment including bevacizumab, the expression level of miR-181a showed a 1.87-fold increase in good responders compared with poor responders^31^. These studies have highlighted the promoting effect of miR-181a on the proliferation, invasion, and migration of tumor cells. Here, we identified the miR-181a-induced effects on CRC angiogenesis. The important findings of our study are as follows: First, miR-181a can induce the ability of tumor cells to promote capillary tube formation of HUVECs. Second, miR-181a is able to promote tumor angiogenesis in nude mice inoculated with CRC cells. Third, miR-181a induces the expression and secretion of VEGF. Finally, bevacizumab, a commonly used anti-VEGF antibody, disrupts the proangiogenic effect of miR-181a, verifying the important role of VEGF in the phenotypic changes induced by miR-181a. However, VEGF is not a direct target gene of miR-181a. Therefore, it is crucial to elucidate the mechanisms by which miR-181a expression in tumor cells affects VEGF expression.

Based on the large sample analysis in the YM500 database, the correlation analysis in both CRC tissues and cell lines, and the *in vitro* and *in vivo* functional studies, we confirmed that miR-181a upregulation potentially causes the downregulation of SRCIN1 expression, and in turn, increases angiogenesis in CRC. Then, clinical samples were tested, and the inverse correlation of miR-181a and SRCIN1 was verified. This correlation was further verified by evaluating the effects of up- and downregulation of miR-181a on SRCIN1 protein expression in CRC cells. Moreover, the effect of SRCIN1 downregulation in CRC cells was similar to the effect of miR-181a overexpression in CRC cells, and the overexpression of SRCIN1 antagonized the effects of miR-181a. This finding suggests that SRCIN1 serves as a downstream mediator of miR-181a function in CRC angiogenesis.

SRCIN1 is widely expressed in normal tissues, such as the mammary glands, lungs, colon, and kidneys^32^. As a tumor suppressor factor, SRCIN1 activates SRC tyrosine kinase (Csk) to inhibit SRC activation in tumor cells^18,33^. SRC belongs to a family of non-receptor tyrosine kinase proteins and plays a key role in angiogenesis via regulation of the gene expression of angiogenic growth factors, such as VEGF^34^. Phosphorylation at Tyr416 can upregulate SRC activity, whereas Csk can phosphorylate at Tyr527 to reduce enzyme activity^35^. However, the role of SRCIN1 in angiogenesis was unclear. In a model of hypoxia, phosphorylation of SRC at Tyr416 induces VEGF expression^36^. Another study showed that SRC-STAT3 signaling leads to VEGF secretion and angiogenesis^37^. In this study, we showed that SRCIN1 can disrupt angiogenesis and inhibit the SRC/VEGF pathway. Therefore, SRCIN1 was confirmed as a functional target of miR-181a and has a vital role in angiogenesis.

Here, we demonstrated that SRCIN1–SRC–VEGF cascade was one of the most important pathway to control angiogenesis in tumor, and blocked this pathway could significantly inhibit angiogenesis. As upregulation of the proangiogenic signaling cascades has been shown to play a crucial role in angiogenesis, the global antiangiogenic effect of SRC inhibitor represents a promising approach for targeting tumor angiogenesis^38^. In this study, we showed that miR-181a can mediate CRC cell communication with endothelial cells to promote angiogenesis through SRCIN1–SRC-VEGF pathway. To date, the biological function of miR-181a has been reported predominantly in the areas of CRC proliferation and liver metastasis through direct targeting of PTEN/AKT, Wnt/β-catenin, PTEN, and WIF-1^13,39–41^. The survey of the particular role of miR-181a in regulating tumor angiogenesis is thus warranted. The identification of miR-181a as a novel angiogenic upstream target of VEGF is thus of high importance, and downregulation of miR-181a may be been developed to a new strategy for CRC treatment. Given the ability of miR-181a to globally promote the angiogenic pathway, it provides a central node to potently block tumor angiogenesis. The colorectal carcinoma is a highly angiogenic cancer type, with high miR-181a levels correlating with significantly deteriorated patient survival. Our complete understanding of the key role of miR-181a in angiogenesis will open rational avenues for therapeutic interventions and promising therapeutic targets in CRC.

In summary, this study demonstrated that miR-181a targets SRCIN1 to promote CRC angiogenesis, and we further identified the miR-181a–SRCIN1–SRC–VEGF axis in CRC (the working model is shown in Fig. 6f). These data confirm the important role of miR-181a in CRC angiogenesis and present a novel therapeutic target for CRC therapy.

## Materials and methods

### Human tissue samples

Human CRC tissues and paired adjacent non-cancerous colorectal tissues were obtained from Tianjin Medical University Cancer Institute and Hospital (Tianjin, China). Written consent was provided by all the patients (or their guardians), and the Ethics Committee of Tianjin Medical University Cancer Institute and Hospital approved all aspects of this study. Tissue fragments were saved in liquid nitrogen at the time of surgery and stored at −80 °C. The clinical features of the patients are listed in supplementary Table 3.

### Cell culture

HT29, SW480, and HUVECs were purchased from the Shanghai Institute of Cell Biology of the Chinese Academy of Sciences (Shanghai, China). HT29 and SW480 cells were cultured in RPMI 1640 medium (Gibco, Carlsbad, CA, USA) supplemented with 10% fetal bovine serum (Gibco) in a humidified incubator at 37 °C in 5% CO_2_. HUVECs were cultured in MCDB 131 medium (Gibco) supplemented with 5% Microvascular Growth Supplement (Gibco) and 1% Gluta MAX (Gibco) in a humidified incubator at 37 °C in 5% CO_2_.

### RNA isolation and quantitative real-time polymerase chain reaction (qRT-PCR)

Total RNA was extracted from the cultured cells and tissues by using TRIzol reagent (Invitrogen, CA, USA). MiR-181a was quantified on an Applied Biosystems 7500 Sequence Detection System by using TaqMan miRNA Assay Probes (Applied Biosystems, Foster City, CA). U6 snRNA was used as the internal control. The relative expression of miR-181a normalized to U6 expression was calculated with the equation 2^−ΔΔCT^, in which ΔΔCT = (CT _miR-181a_ − CT _U6_) tumor− (CT _miR-181a_ − CT _U6_) control.

To quantify SRCIN1 and GAPDH mRNA expression, oligo d (T) 18 primers (TaKaRa) were used to reverse transcribe total RNA into cDNA. Then, qRT-PCR was performed by using SYBR Green dye (Invitrogen) and specific primers for SRCIN1 and GAPDH. The primer sequences were as follows: SRCIN1, (sense) 5′-GAACGGCTGCGCTATCTCAA-3′ and (antisense) 5′-GGATCTTCTCCACCGATTTCTCC-′; and VEGF, (sense) 5′-CTTGCCTTGCTGCTCTACCT-3′ (antisense)5′-GATTCTGCCCTCCTCCTTCT-3′ and GAPDH, (sense) 5′-CGAGCCACATCGCTCAGACA-3′ and (antisense) 5′ GTGGTGAAGACGCCAG-3′

### miRNA expression analysis

For the microarray analysis, independent pooled tissue samples were analyzed from CRC and NAT. Each sample comprised a pool of tissues from five clinical samples. Total RNA from each pooled sample was isolated using the TRIzol method for Affymetrix miRNA microarray analysis (CapitalBio Corp, Beijing, China). Procedures were performed as described on the web site of CapitalBio (http://www.capitalbio.com). In brief, 50–100 mg of total RNA was used to extract miRNA with a miRNA Isolation Kit (Ambion Inc., Texas, USA). Biotin-labeled miRNAs were used for hybridization on each miRNA microarray chip containing probes. Raw data were normalized to U6 and analyzed using GenePix Pro 4.0 software (Axon Instruments, PA, USA).

### Western blot

Radioimmunoprecipitation lysis buffer (Beyotime, Shanghai, China) freshly mixed with a protease and phosphatase inhibitor cocktail (Thermo Scientific, Rockford, Cambridge, MA) was used to isolate proteins from cells or tissues. Proteins were separated on 8% or 10% SDS-PAGE gels (Bio-Rad). Antibodies for western blotting were as follows: anti-SNIP25 interacting protein antibody (Abcam ab111343, USA), anti-phospho-SRC family (Y416) and anti-phospho-SRC (Y527) were purchased from Cell Signaling Technology (#6943 and #2105, respectively; CST,USA), antibodies against SRC, VEGF and GAPDH were purchased from Santa Cruz Biotechnology (sc-8056, sc-152, and sc-365062, respectively; Santa Cruz, CA, USA).

### Overexpression or knockdown of miR-181a

Synthetic miR-181a mimic and inhibitor and corresponding negative control RNA were purchased from Ribobio (Guangzhou, China). CRC cells were transfected with RNA oligoribonucleotides using Lipofectamine 2000 (Invitrogen, CA, USA). Generally, 100 pmol of miR-181a mimic, inhibitor, or scrambled negative control RNAs was used for each transfection. After 48 h, the transfected cells were harvested for the following experiments.

### Overexpression or knockdown of SRCIN1

A vector encoding the human SRCIN1 open reading frame without the 3′-UTR (EX-Y4423-M68) was obtained from GeneCopoeia (Germantown, MD, USA), and an empty plasmid served as the negative control. The siRNA sequence (5′-AAGCTGTGTCTGTTGAGGCTG-3′) targeting human SRCIN1 was synthesized by RiboBio (Guangzhou, China), and a scrambled siRNA (RiboBio) was used as the negative control.

### Luciferase reporter assay

The SRCIN1 3′-UTR WT vector was constructed by inserting the amplified 3′-UTR of human SRCIN1 into a luciferase reporter vector. The sequences interacting with the miR-181a seed sequence were mutated from TGAATGT to ACTTACA, and the mutant SRCIN1 3′-UTR was inserted into an equivalent luciferase reporter plasmid to construct the SRCIN1 3′-UTR-MUT vector. Luciferase reporter plasmid (1 μg), β-galactosidase (β-gal) expression plasmid (1 μg), and equal amounts (100 pmol) of the miR-181a mimic, inhibitor, or scrambled negative control RNA were cotransfected into CRC cells using Lipofectamine 2000, and the β-gal plasmid was used as a transfection control. After 24 h, the cells were analyzed for luciferase activity by using luciferase assay kits (Promega, Madison, WI, USA) and a Modulus Luminometer (Turner Biosystems, Sunnyvale, USA).

### ELISA

The concentration of VEGF in cell culture medium from HT29 or SW480 cells was determined by enzyme-linked immunosorbent assay by using a human VEGF ELISA kit (4A Biotech, Beijing, China). The assay was performed following the manufacturer’s instructions.

### Preparation of TCM

Tumor cells (1 × 10^5^) were transfected with or without DNA or RNA in a 12-well plate with RPMI 1640 medium supplemented with 2% fetal bovine serum. Eighteen hours after transfection, the medium was collected and centrifuged at 500 g to remove cells; then, the supernatant was further centrifuged at 12,000 g to discard the cell debris.

### Capillary tube formation assay

*In vitro* angiogenesis (capillary tube formation assay) was performed as previously described^42,43^. In brief, 200 μl of Matrigel (BD Biosciences Pharmingen, San Diago, CA, USA) was added to each well of a 24-well plate and allowed to polymerize at 37°C for 30 min. Before the capillary tube formation assay, to serum-starve HUVECs, the cells were incubated in MCDB 131 media containing 1% microvascular growth supplement for 8 h. HUVECs (5 × 10^4^) were grown in TCM in a coated plate at 37°C. After 6 h, the HUVECs were photographed to assess the formation of capillary-like structures. The number of branches represented the degree of *in vitro* angiogenesis. Bevacizumab (4 ml at 25 mg/ml) was obtained from Tianjin Medical University Cancer Institute and Hospital.

### *In Vivo* Matrigel Plug Angiogenesis Assay

All animal experimental procedures were approved by the Institutional Animal Care and Research Advisory Committee of Nanjing University. Matrigel plug assays were conducted as described^42,43^. In brief, 1 × 10^7^ HT29-miR-181a or HT29-control cells premixed with growth factor-reduced Matrigel (500 μl, cat. 3433-005-01, R&D Systems, Minnesota, USA) were subcutaneously implanted into the flanks of the same nude mouse (BALB/c nu/nu, 6 weeks old), with each cell type implanted on a different side. Six nude mice were included in the study. After 7 days, the animals were killed, and the Matrigel plugs were removed, embedded in Opti-mum Cutting Temperature (OCT) (Miles, Elkhart, IN), and stored at −80 °C.

### IHC staining

Matrigel plug sections and paraffin-embedded tissue sections were used for IHC staining using CD34 antibody (cat. sc-52312, Santa Cruz Biotechnology, Santa Cruz, CA) or mAb for VEGF (sc-152, Santa Cruz), or Ki-67 (Abcam, ab16667). The microvessel density in the tumor tissues or Matrigel plug, which represents the degree of angiogenesis *in vivo*, was evaluated by staining for CD34 (a biomarker of microvessel density). Any discrete cluster or single cell stained with CD34 was counted as one microvessel.

### Fluorescence in situ hybridization (FISH) and immunofluorescence (IF)

FISH of miR-181a was performed using a 5′-DIG-and 3′-DIG-labeled miRCURY LNA Detection Probe (Exiqon, Vedbaek, Denmark). The sequence of the miR-181a probe was as follow: 5′ Dig-ACTCACCGACAGCGTTGAATGTT-Dig 3′. In brief, after deparaffinised and rehydrated, the tissue sections were digested with proteinase K (20 μg/ml, 37 °C for 10 min), and hybridized overnight at 37 °C with the above mentioned probe. Finally, the anti-digoxigenin antibody was used to visualize the positive hybridization signals. Finally, FISH of the tissue sections were visualized using fluorescent microscopy (Nikon, Eclipse CI, Tokyo, Japan).

The tissue sections were fixed in 4% paraformaldehyde for 30 min at room temperature (RT). After fixation, the tissue sections were washed with phosphate-buffered saline (PBS) (3 × 5 min, RT), and then permeabilized and blocked using 5% bovine serum albumin (BSA) (Sigma, St Louis, MO) and 0.5% Triton X-100 in PBS for 1 hat RT. Next, the tissue sections were incubated with primary antibody for SRCIN1 (Abcam ab111343) in 5% BSA overnight at 4 °C, and then rinsed in PBS (3 × 5 min, RT). The tissue sections were then incubated in secondary fluorescent antibody (Invitrogen, 594 nm) in 5% BSA in a light-proof environment for 1 h, at RT. Next, the tissue sections were stained with DAPI (Beyotime) a light-proof environment for 10 min at RT. Finally, IF of the tissue sections were visualized using fluorescent microscopy (Nikon, Eclipse CI, Tokyo, Japan).

### Statistical analysis

All data are expressed as the means ± SD. The *P* value of the difference between groups was measured using Student’s t-test for comparisons of two groups or one-way analysis of variance for multiple comparisons. A *P* value < 0.05 was considered statistically significant (*P < 0.05, **P < 0.01, ***P < 0.001).

## Electronic supplementary material

Suppementary information

Table S1

Table S2

Table S3

Supplementary Figure 1

Supplementary Figure 2

Supplementary Figure 3

Supplementary Figure 4

Supplementary Figure 5

Supplementary Figure 6
