# Supplementary material for: MicroRNA-181a promotes angiogenesis in colorectal cancer by targeting SRCIN1 to promote the SRC/VEGF signaling pathway
Source: Cell Death Dis. 2018 Apr 19;9(4):438. doi: 10.1038/s41419-018-0490-4 (PMC5941226; doi:10.1038/s41419-018-0490-4)
Supplement: Supplementary file 1 — Suppementary information [file 41419_2018_490_MOESM1_ESM.docx]

**Supplementary Table 1.** MiRNAs differentially expressed in CRC

**Supplementary Table 2.** Meta-analysis of the miR-181a and SRCIN1 expression in patterns of 8 normal solid tissues and 457 colon adenocarcinoma

**Supplementary Table 3.** Patients’ Characteristics

**Supplementary Figure 1.** (A) Venn diagram of two chips showing the overlapped miRNA from two studies (A: our current study: B: our previous report (reference 19)). (B) Biological pathway analysis of miRNAs by FunRich.

**Supplementary Figure 2.** Supplemental data of miR-181a in SW480.(a) Quantitative RT-PCR analysis of miR-181a expression levels in SW480 cells transfected with control mimic, miR-181a mimic, control inhibitor or miR-181a inhibitor.(b and c) HUVECs were cultured in the presence of 75% TCM from SW480 cells transfected with control mimic, miR-181a mimic, control inhibitor or miR-181a inhibitor, scale bars: 100 μm; (b) representative images of tube formation; (c) HUVEC branch number. Data are shown as the mean ± SD of three replicates.**P* < 0.05; ***P* < 0.01; ****P* < 0.001.

**Supplementary Figure 3.** (a and b )Infection efficiency of lentivirus vectors containing NC or miR-181a, scale bars: 100 μm; (a) representative images; (b) quantitative RT-PCR analysis. Data are shown as the mean ± SD of three replicates. ***P* < 0.01.

**Supplementary Figure 4.** Tumors from implanted mice were subjected to H&E-stained, scale bars: 50 μm

**Supplementary Figure 5.** (a)Quantitative RT-PCR analysis of SRCIN1 mRNA expression levels in HT29 cells transfected with control mimic, miR-181a mimic, control inhibitor or miR-181a inhibitor. (b)Quantitative RT-PCR analysis of VEGF mRNA expression levels in HT29 cells transfected with control mimic, miR-181a mimic, control inhibitor or miR-181a inhibitor. (c)Quantitative RT-PCR analysis of SRCIN1 mRNA expression levels in SW480 cells transfected with control mimic, miR-181a mimic, control inhibitor or miR-181a inhibitor. (d)Quantitative RT-PCR analysis of VEGF mRNA expression levels in SW480 cells transfected with control mimic, miR-181a mimic, control inhibitor or miR-181a inhibitor. Data are shown as the mean ± SD of three replicates. **P* < 0.05; ***P* < 0.01.

**Supplementary Figure 6.** (a) Quantitative RT-PCR analysis of SRCIN1 mRNA expression levels in HT29 cells transfected with control vector, SRCIN1 vector control siRNA or si-SRCIN1. (b)Quantitative RT-PCR analysis of VEGF mRNA expression levels in HT29 cells transfected with control vector, SRCIN1 vector control siRNA or si-SRCIN1. Data are shown as the mean ± SD of three replicates. **P* < 0.05; ***P* < 0.01.
