# Supplementary material for: MicroRNA-181a promotes angiogenesis in colorectal cancer by targeting SRCIN1 to promote the SRC/VEGF signaling pathway
Source: Cell Death Dis. 2018 Apr 19;9(4):438. doi: 10.1038/s41419-018-0490-4 (PMC5941226; doi:10.1038/s41419-018-0490-4)
Supplement: Supplementary file 2 — Table S1 [file 41419_2018_490_MOESM2_ESM.docx]

**Supplementary Table 1. miRNAs differentially expressed in CRC**

**Upregulated miRNAs(39)**

| \| \| Gene ID \| \|  \| \| CRC \|  \| \| \| N \|  \| \| fold change \| \| \| --- \| --- \| --- \| --- \| --- \| --- \| --- \| --- \| --- \| --- \| --- \| --- \| --- \| \| hsa-let-7e-5p \|  \| \| 2380.958 \| \| \|  \| 136.4581 \| \| \|  \| \| 17.4483 \| \| hsa-miR-100-5p \|  \| \| 1684.521 \| \| \|  \| 135.8051 \| \| \|  \| \| 12.404 \| \| hsa-miR-143-3p \|  \| \| 9619.338 \| \| \|  \| 1200.365 \| \| \|  \| \| 8.0137 \| \| hsa-miR-125b-5p \|  \| \| 3379.958 \| \| \|  \| 449.9585 \| \| \|  \| \| 7.5117 \| \| hsa-miR-145-5p \|  \| \| 14416.16 \| \| \|  \| 2144.281 \| \| \|  \| \| 6.7231 \| \| hsa-miR-27b-3p \|  \| \| 1932.232 \| \| \|  \| 344.1487 \| \| \|  \| \| 5.6145 \| \| hsa-miR-195-5p \|  \| \| 1801.116 \| \| \|  \| 326.8746 \| \| \|  \| \| 5.5101 \| \| hsa-miR-125a-5p \|  \| \| 2208.137 \| \| \|  \| 408.955 \| \| \|  \| \| 5.3995 \| \| hsa-miR-23b-3p \|  \| \| 7524.064 \| \| \|  \| 1400.617 \| \| \|  \| \| 5.372 \| \| hsa-miR-574-3p \|  \| \| 791.6973 \| \| \|  \| 155.2954 \| \| \|  \| \| 5.098 \| \| hsa-miR-99b-5p \|  \| \| 1177.628 \| \| \|  \| 237.0308 \| \| \|  \| \| 4.9682 \| \| hsa-miR-214-3p \|  \| \| 757.1343 \| \| \|  \| 179.62 \| \| \|  \| \| 4.2152 \| \| hsa-miR-4454 \|  \| \| 976.9587 \| \| \|  \| 238.9582 \| \| \|  \| \| 4.0884 \| \| hsa-miR-30c-5p \|  \| \| 659.9583 \| \| \|  \| 168.4582 \| \| \|  \| \| 3.9176 \| \| hsa-miR-28-5p \|  \| \| 675.024 \| \| \|  \| 173.0128 \| \| \|  \| \| 3.9016 \| \| hsa-miR-152-3p \|  \| \| 428.6675 \| \| \|  \| 121.3555 \| \| \|  \| \| 3.5323 \| \| hsa-miR-199a-5p \|  \| \| 447.506 \| \| \|  \| 134.4212 \| \| \|  \| \| 3.3291 \| \| hsa-miR-193b-3p \|  \| \| 426.8832 \| \| \|  \| 138.5792 \| \| \|  \| \| 3.0804 \| \| hsa-miR-26a-5p \|  \| \| 6833.573 \| \| \|  \| 2409.602 \| \| \|  \| \| 2.836 \| \| hsa-miR-28-3p \|  \| \| 419.0447 \| \| \|  \| 147.8427 \| \| \|  \| \| 2.8344 \| \| hsa-miR-10a-5p \|  \| \| 500.6693 \| \| \|  \| 177.5203 \| \| \|  \| \| 2.8203 \| \| hsa-miR-422a \|  \| \| 310.4708 \| \| \|  \| 113.0423 \| \| \|  \| \| 2.7465 \| \| hsa-miR-378i \|  \| \| 327.1013 \| \| \|  \| 119.177 \| \| \|  \| \| 2.7447 \| \| hsa-let-7f-5p \|  \| \| 844.3084 \| \| \|  \| 314.0307 \| \| \|  \| \| 2.6886 \| \| hsa-miR-27a-3p \|  \| \| 1636.46 \| \| \|  \| 621.2639 \| \| \|  \| \| 2.6341 \| \| hsa-miR-24-3p \|  \| \| 6019.666 \| \| \|  \| 2289.438 \| \| \|  \| \| 2.6293 \| \| hsa-miR-22-3p \|  \| \| 1125.459 \| \| \|  \| 444.4583 \| \| \|  \| \| 2.5322 \| \| hsa-let-7d-5p \|  \| \| 3510.702 \| \| \|  \| 1427.377 \| \| \|  \| \| 2.4595 \| \| hsa-miR-378a-3p \|  \| \| 1681.159 \| \| \|  \| 710.6154 \| \| \|  \| \| 2.3658 \| \| hsa-miR-199a-3p \|  \| \| 1382.861 \| \| \|  \| 604.5303 \| \| \|  \| \| 2.2875 \| \| hsa-miR-199b-3p \|  \| \| 1382.861 \| \| \|  \| 604.5303 \| \| \|  \| \| 2.2875 \| \| hsa-miR-320a \|  \| \| 1917.421 \| \| \|  \| 873.1365 \| \| \|  \| \| 2.196 \| \| hsa-miR-320b \|  \| \| 1794.201 \| \| \|  \| 828.4512 \| \| \|  \| \| 2.1657 \| \| hsa-let-7a-5p \|  \| \| 6391.958 \| \| \|  \| 2952.957 \| \| \|  \| \| 2.1646 \| \| hsa-miR-378f \|  \| \| 358.1215 \| \| \|  \| 168.1567 \| \| \|  \| \| 2.1297 \| \| hsa-miR-181a-5p \|  \| \| 1012.446 \| \| \|  \| 479.8091 \| \| \|  \| \| 2.1101 \| \| hsa-miR-140-3p \|  \| \| 413.1299 \| \| \|  \| 199.1618 \| \| \|  \| \| 2.0743 \| \| hsa-miR-378c \|  \| \| 650.5961 \| \| \|  \| 313.667 \| \| \|  \| \| 2.0742 \| \| hsa-miR-320c \|  \| \| 1693.848 \| \| \|  \| 818.1539 \| \| \|  \| \| 2.0703 \| \| \| --- \| --- \| --- \| --- \| --- \| --- \| --- \| --- \| --- \| --- \| --- \| --- \| --- \| --- \| --- \| --- \| --- \| --- \| --- \| --- \| --- \| --- \| --- \| --- \| --- \| --- \| --- \| --- \| --- \| --- \| --- \| --- \| --- \| --- \| --- \| --- \| --- \| --- \| --- \| --- \| --- \| --- \| --- \| --- \| --- \| --- \| --- \| --- \| --- \| --- \| --- \| --- \| --- \| --- \| --- \| --- \| --- \| --- \| --- \| --- \| --- \| --- \| --- \| --- \| --- \| --- \| --- \| --- \| --- \| --- \| --- \| --- \| --- \| --- \| --- \| --- \| --- \| --- \| --- \| --- \| --- \| --- \| --- \| --- \| --- \| --- \| --- \| --- \| --- \| --- \| --- \| --- \| --- \| --- \| --- \| --- \| --- \| --- \| --- \| --- \| --- \| --- \| --- \| --- \| --- \| --- \| --- \| --- \| --- \| --- \| --- \| --- \| --- \| --- \| --- \| --- \| --- \| --- \| --- \| --- \| --- \| --- \| --- \| --- \| --- \| --- \| --- \| --- \| --- \| --- \| --- \| --- \| --- \| --- \| --- \| --- \| --- \| --- \| --- \| --- \| --- \| --- \| --- \| --- \| --- \| --- \| --- \| --- \| --- \| --- \| --- \| --- \| --- \| --- \| --- \| --- \| --- \| --- \| --- \| --- \| --- \| --- \| --- \| --- \| --- \| --- \| --- \| --- \| --- \| --- \| --- \| --- \| --- \| --- \| --- \| --- \| --- \| --- \| --- \| --- \| --- \| --- \| --- \| --- \| --- \| --- \| --- \| --- \| --- \| --- \| --- \| --- \| --- \| --- \| --- \| --- \| --- \| --- \| --- \| --- \| --- \| --- \| --- \| --- \| --- \| --- \| --- \| --- \| --- \| --- \| --- \| --- \| --- \| --- \| --- \| --- \| --- \| --- \| --- \| --- \| --- \| --- \| --- \| --- \| --- \| --- \| --- \| --- \| --- \| --- \| --- \| --- \| --- \| --- \| --- \| --- \| --- \| --- \| --- \| --- \| --- \| --- \| --- \| --- \| --- \| --- \| --- \| --- \| --- \| --- \| --- \| --- \| --- \| --- \| --- \| --- \| --- \| --- \| --- \| --- \| --- \| --- \| --- \| --- \| --- \| --- \| --- \| --- \| --- \| --- \| --- \| --- \| --- \| --- \| --- \| --- \| --- \| --- \| --- \| --- \| --- \| --- \| --- \| --- \| --- \| --- \| --- \| --- \| --- \| --- \| --- \| --- \| --- \| --- \| --- \| --- \| --- \| --- \| --- \| --- \| --- \| --- \| --- \| --- \| --- \| --- \| --- \| --- \| --- \| --- \| --- \| --- \| --- \| --- \| --- \| --- \| --- \| --- \| --- \| --- \| --- \| --- \| --- \| --- \| --- \| --- \| --- \| --- \| --- \| --- \| --- \| --- \| --- \| --- \| --- \| --- \| --- \| --- \| --- \| --- \| --- \| --- \| --- \| --- \| --- \| --- \| --- \| --- \| --- \| --- \| --- \| --- \| --- \| --- \| --- \| --- \| --- \| --- \| --- \| --- \| --- \| --- \| --- \| --- \| --- \| --- \| --- \| --- \| --- \| --- \| --- \| --- \| --- \| --- \| --- \| --- \| --- \| --- \| --- \| --- \| --- \| --- \| --- \| --- \| --- \| --- \| --- \| --- \| --- \| --- \| --- \| --- \| --- \| --- \| --- \| --- \| --- \| --- \| --- \| --- \| --- \| --- \| --- \| --- \| --- \| --- \| --- \| --- \| --- \| --- \| --- \| --- \| --- \| --- \| --- \| --- \| --- \| --- \| --- \| --- \| --- \| --- \| --- \| --- \| --- \| --- \| --- \| --- \| --- \| --- \| --- \| --- \| --- \| --- \| --- \| --- \| --- \| --- \| --- \| --- \| --- \| --- \| --- \| --- \| --- \| --- \| --- \| --- \| --- \| --- \| --- \| --- \| --- \| --- \| --- \| --- \| --- \| --- \| --- \| --- \| --- \| --- \| --- \| --- \| --- \| --- \| --- \| --- \| --- \| --- \| --- \| --- \| --- \| --- \| --- \| --- \| --- \| --- \| --- \| --- \| --- \| --- \| --- \| --- \| --- \| --- \| --- \| --- \| --- \| --- \| --- \| --- \| --- \| --- \| --- \| --- \| --- \| --- \| --- \| --- \| --- \| --- \| --- \| --- \| --- \| --- \| --- \| --- \| --- \| --- \| --- \| --- \| --- \| --- \| --- \| --- \| --- \| --- \| --- \| --- \| --- \| |
| --- | --- | --- | --- | --- | --- | --- | --- | --- | --- | --- | --- | --- | --- | --- | --- | --- | --- | --- | --- | --- | --- | --- | --- | --- | --- | --- | --- | --- | --- | --- | --- | --- | --- | --- | --- | --- | --- | --- | --- | --- | --- | --- | --- | --- | --- | --- | --- | --- | --- | --- | --- | --- | --- | --- | --- | --- | --- | --- | --- | --- | --- | --- | --- | --- | --- | --- | --- | --- | --- | --- | --- | --- | --- | --- | --- | --- | --- | --- | --- | --- | --- | --- | --- | --- | --- | --- | --- | --- | --- | --- | --- | --- | --- | --- | --- | --- | --- | --- | --- | --- | --- | --- | --- | --- | --- | --- | --- | --- | --- | --- | --- | --- | --- | --- | --- | --- | --- | --- | --- | --- | --- | --- | --- | --- | --- | --- | --- | --- | --- | --- | --- | --- | --- | --- | --- | --- | --- | --- | --- | --- | --- | --- | --- | --- | --- | --- | --- | --- | --- | --- | --- | --- | --- | --- | --- | --- | --- | --- | --- | --- | --- | --- | --- | --- | --- | --- | --- | --- | --- | --- | --- | --- | --- | --- | --- | --- | --- | --- | --- | --- | --- | --- | --- | --- | --- | --- | --- | --- | --- | --- | --- | --- | --- | --- | --- | --- | --- | --- | --- | --- | --- | --- | --- | --- | --- | --- | --- | --- | --- | --- | --- | --- | --- | --- | --- | --- | --- | --- | --- | --- | --- | --- | --- | --- | --- | --- | --- | --- | --- | --- | --- | --- | --- | --- | --- | --- | --- | --- | --- | --- | --- | --- | --- | --- | --- | --- | --- | --- | --- | --- | --- | --- | --- | --- | --- | --- | --- | --- | --- | --- | --- | --- | --- | --- | --- | --- | --- | --- | --- | --- | --- | --- | --- | --- | --- | --- | --- | --- | --- | --- | --- | --- | --- | --- | --- | --- | --- | --- | --- | --- | --- | --- | --- | --- | --- | --- | --- | --- | --- | --- | --- | --- | --- | --- | --- | --- | --- | --- | --- | --- | --- | --- | --- | --- | --- | --- | --- | --- | --- | --- | --- | --- | --- | --- | --- | --- | --- | --- | --- | --- | --- | --- | --- | --- | --- | --- | --- | --- | --- | --- | --- | --- | --- | --- | --- | --- | --- | --- | --- | --- | --- | --- | --- | --- | --- | --- | --- | --- | --- | --- | --- | --- | --- | --- | --- | --- | --- | --- | --- | --- | --- | --- | --- | --- | --- | --- | --- | --- | --- | --- | --- | --- | --- | --- | --- | --- | --- | --- | --- | --- | --- | --- | --- | --- | --- | --- | --- | --- | --- | --- | --- | --- | --- | --- | --- | --- | --- | --- | --- | --- | --- | --- | --- | --- | --- | --- | --- | --- | --- | --- | --- | --- | --- | --- | --- | --- | --- | --- | --- | --- | --- | --- | --- | --- | --- | --- | --- | --- | --- | --- | --- | --- | --- | --- | --- | --- | --- | --- | --- | --- | --- | --- | --- | --- | --- | --- | --- | --- | --- | --- | --- | --- | --- | --- | --- | --- | --- | --- | --- | --- | --- | --- | --- | --- | --- | --- | --- | --- | --- | --- | --- | --- | --- | --- | --- | --- | --- | --- | --- | --- | --- | --- | --- | --- | --- | --- | --- | --- | --- | --- | --- | --- | --- | --- | --- | --- | --- | --- | --- | --- | --- | --- | --- | --- | --- | --- | --- | --- | --- | --- | --- |

**Downregulated miRNAs (48)**

| \| \| Gene ID \| \|  \| \| CRC \|  \| \| N \|  \| \| fold change \| \| \| --- \| --- \| --- \| --- \| --- \| --- \| --- \| --- \| --- \| --- \| --- \| --- \| \| hsa-miR-3621 \|  \| \| 142.0714 \| \|  \| 286.8616 \| \| \|  \| \| 0.4953 \| \| hsa-miR-6782-5p \|  \| \| 49.64536 \| \|  \| 100.4285 \| \| \|  \| \| 0.4943 \| \| hsa-miR-1908-5p \|  \| \| 946.6274 \| \|  \| 1923.493 \| \| \|  \| \| 0.4921 \| \| hsa-miR-19b-3p \|  \| \| 324.4581 \| \|  \| 659.9578 \| \| \|  \| \| 0.4916 \| \| hsa-miR-1909-3p \|  \| \| 134.4757 \| \|  \| 273.8003 \| \| \|  \| \| 0.4911 \| \| hsa-miR-200b-5p \|  \| \| 93.11417 \| \|  \| 190.1639 \| \| \|  \| \| 0.4897 \| \| hsa-miR-5196-5p \|  \| \| 56.95817 \| \|  \| 117.4582 \| \| \|  \| \| 0.4849 \| \| hsa-miR-6765-5p \|  \| \| 502.2538 \| \|  \| 1046.713 \| \| \|  \| \| 0.4798 \| \| hsa-miR-20a-5p \|  \| \| 881.2428 \| \|  \| 1837.872 \| \| \|  \| \| 0.4795 \| \| hsa-miR-146a-5p \|  \| \| 405.5185 \| \|  \| 851.316 \| \| \|  \| \| 0.4763 \| \| hsa-miR-3141 \|  \| \| 367.5528 \| \|  \| 776.8411 \| \| \|  \| \| 0.4731 \| \| hsa-miR-92a-3p \|  \| \| 2430.957 \| \|  \| 5149.462 \| \| \|  \| \| 0.4721 \| \| hsa-miR-6778-5p \|  \| \| 78.47417 \| \|  \| 166.2238 \| \| \|  \| \| 0.4721 \| \| hsa-miR-6769b-5p \|  \| \| 56.82557 \| \|  \| 120.5518 \| \| \|  \| \| 0.4714 \| \| hsa-miR-328-5p \|  \| \| 466.3412 \| \|  \| 1029.111 \| \| \|  \| \| 0.4531 \| \| hsa-miR-3180-3p \|  \| \| 82.80135 \| \|  \| 183.1986 \| \| \|  \| \| 0.452 \| \| hsa-miR-4649-5p \|  \| \| 152.1708 \| \|  \| 339.6088 \| \| \|  \| \| 0.4481 \| \| hsa-miR-664b-3p \|  \| \| 51.95816 \| \|  \| 119.4582 \| \| \|  \| \| 0.4349 \| \| hsa-miR-155-5p \|  \| \| 829.1119 \| \|  \| 1938.436 \| \| \|  \| \| 0.4277 \| \| hsa-miR-1343-5p \|  \| \| 167.7154 \| \|  \| 399.284 \| \| \|  \| \| 0.42 \| \| hsa-miR-200a-3p \|  \| \| 126.9255 \| \|  \| 305.2848 \| \| \|  \| \| 0.4158 \| \| hsa-miR-7150 \|  \| \| 79.86703 \| \|  \| 192.7573 \| \| \|  \| \| 0.4143 \| \| hsa-miR-3175 \|  \| \| 96.66911 \| \|  \| 234.3625 \| \| \|  \| \| 0.4125 \| \| hsa-miR-4449 \|  \| \| 92.96742 \| \|  \| 226.8212 \| \| \|  \| \| 0.4099 \| \| hsa-miR-885-3p \|  \| \| 74.61849 \| \|  \| 183.3945 \| \| \|  \| \| 0.4069 \| \| hsa-miR-6805-5p \|  \| \| 510.4323 \| \|  \| 1258.341 \| \| \|  \| \| 0.4056 \| \| hsa-miR-6836-5p \|  \| \| 237.101 \| \|  \| 588.5624 \| \| \|  \| \| 0.4028 \| \| hsa-miR-8072 \|  \| \| 1290.643 \| \|  \| 3218.218 \| \| \|  \| \| 0.401 \| \| hsa-miR-6787-5p \|  \| \| 143.2904 \| \|  \| 366.2533 \| \| \|  \| \| 0.3912 \| \| hsa-miR-3180 \|  \| \| 62.40949 \| \|  \| 160.6123 \| \| \|  \| \| 0.3886 \| \| hsa-miR-4417 \|  \| \| 121.5301 \| \|  \| 313.792 \| \| \|  \| \| 0.3873 \| \| hsa-miR-4656 \|  \| \| 82.18269 \| \|  \| 214.8173 \| \| \|  \| \| 0.3826 \| \| hsa-miR-200b-3p \|  \| \| 338.2187 \| \|  \| 903.3793 \| \| \|  \| \| 0.3744 \| \| hsa-miR-203a \|  \| \| 126.2125 \| \|  \| 338.386 \| \| \|  \| \| 0.373 \| \| hsa-miR-663a \|  \| \| 289.2959 \| \|  \| 816.7079 \| \| \|  \| \| 0.3542 \| \| hsa-miR-663b \|  \| \| 43.93461 \| \|  \| 124.5922 \| \| \|  \| \| 0.3526 \| \| hsa-miR-665 \|  \| \| 50.46508 \| \|  \| 149.2544 \| \| \|  \| \| 0.3381 \| \| hsa-miR-1233-5p \|  \| \| 143.9824 \| \|  \| 439.9026 \| \| \|  \| \| 0.3273 \| \| hsa-miR-4443 \|  \| \| 134.8337 \| \|  \| 420.472 \| \| \|  \| \| 0.3207 \| \| hsa-miR-1207-5p \|  \| \| 390.3116 \| \|  \| 1293.615 \| \| \|  \| \| 0.3017 \| \| hsa-miR-6848-5p \|  \| \| 30.97805 \| \|  \| 104.4551 \| \| \|  \| \| 0.2966 \| \| hsa-miR-215-5p \|  \| \| 129.9907 \| \|  \| 450.4764 \| \| \|  \| \| 0.2886 \| \| hsa-miR-5739 \|  \| \| 55.50729 \| \|  \| 195.6593 \| \| \|  \| \| 0.2837 \| \| hsa-miR-1246 \|  \| \| 389.9583 \| \|  \| 1408.459 \| \| \|  \| \| 0.2769 \| \| hsa-miR-4467 \|  \| \| 300.7275 \| \|  \| 1164.443 \| \| \|  \| \| 0.2583 \| \| hsa-miR-4728-5p \|  \| \| 39.95816 \| \|  \| 154.9582 \| \| \|  \| \| 0.2579 \| \| hsa-miR-1224-5p \|  \| \| 42.46665 \| \|  \| 175.9229 \| \| \|  \| \| 0.2414 \| \| hsa-miR-18a-5p \|  \| \| 61.9263 \| \|  \| 260.6325 \| \| \|  \| \| 0.2376 \| \| \| --- \| --- \| --- \| --- \| --- \| --- \| --- \| --- \| --- \| --- \| --- \| --- \| --- \| --- \| --- \| --- \| --- \| --- \| --- \| --- \| --- \| --- \| --- \| --- \| --- \| --- \| --- \| --- \| --- \| --- \| --- \| --- \| --- \| --- \| --- \| --- \| --- \| --- \| --- \| --- \| --- \| --- \| --- \| --- \| --- \| --- \| --- \| --- \| --- \| --- \| --- \| --- \| --- \| --- \| --- \| --- \| --- \| --- \| --- \| --- \| --- \| --- \| --- \| --- \| --- \| --- \| --- \| --- \| --- \| --- \| --- \| --- \| --- \| --- \| --- \| --- \| --- \| --- \| --- \| --- \| --- \| --- \| --- \| --- \| --- \| --- \| --- \| --- \| --- \| --- \| --- \| --- \| --- \| --- \| --- \| --- \| --- \| --- \| --- \| --- \| --- \| --- \| --- \| --- \| --- \| --- \| --- \| --- \| --- \| --- \| --- \| --- \| --- \| --- \| --- \| --- \| --- \| --- \| --- \| --- \| --- \| --- \| --- \| --- \| --- \| --- \| --- \| --- \| --- \| --- \| --- \| --- \| --- \| --- \| --- \| --- \| --- \| --- \| --- \| --- \| --- \| --- \| --- \| --- \| --- \| --- \| --- \| --- \| --- \| --- \| --- \| --- \| --- \| --- \| --- \| --- \| --- \| --- \| --- \| --- \| --- \| --- \| --- \| --- \| --- \| --- \| --- \| --- \| --- \| --- \| --- \| --- \| --- \| --- \| --- \| --- \| --- \| --- \| --- \| --- \| --- \| --- \| --- \| --- \| --- \| --- \| --- \| --- \| --- \| --- \| --- \| --- \| --- \| --- \| --- \| --- \| --- \| --- \| --- \| --- \| --- \| --- \| --- \| --- \| --- \| --- \| --- \| --- \| --- \| --- \| --- \| --- \| --- \| --- \| --- \| --- \| --- \| --- \| --- \| --- \| --- \| --- \| --- \| --- \| --- \| --- \| --- \| --- \| --- \| --- \| --- \| --- \| --- \| --- \| --- \| --- \| --- \| --- \| --- \| --- \| --- \| --- \| --- \| --- \| --- \| --- \| --- \| --- \| --- \| --- \| --- \| --- \| --- \| --- \| --- \| --- \| --- \| --- \| --- \| --- \| --- \| --- \| --- \| --- \| --- \| --- \| --- \| --- \| --- \| --- \| --- \| --- \| --- \| --- \| --- \| --- \| --- \| --- \| --- \| --- \| --- \| --- \| --- \| --- \| --- \| --- \| --- \| --- \| --- \| --- \| --- \| --- \| --- \| --- \| --- \| --- \| --- \| --- \| --- \| --- \| --- \| --- \| --- \| --- \| --- \| --- \| --- \| --- \| --- \| --- \| --- \| --- \| --- \| --- \| --- \| --- \| --- \| --- \| --- \| --- \| --- \| --- \| --- \| --- \| --- \| --- \| --- \| --- \| --- \| --- \| --- \| --- \| --- \| --- \| --- \| --- \| --- \| --- \| --- \| --- \| --- \| --- \| --- \| --- \| --- \| --- \| --- \| --- \| --- \| --- \| --- \| --- \| --- \| --- \| --- \| --- \| --- \| --- \| --- \| --- \| --- \| --- \| --- \| --- \| --- \| --- \| --- \| --- \| --- \| --- \| --- \| --- \| --- \| --- \| --- \| --- \| --- \| --- \| --- \| --- \| --- \| --- \| --- \| --- \| --- \| --- \| --- \| --- \| --- \| --- \| --- \| --- \| --- \| --- \| --- \| --- \| --- \| --- \| --- \| --- \| --- \| --- \| --- \| --- \| --- \| --- \| --- \| --- \| --- \| --- \| --- \| --- \| --- \| --- \| --- \| --- \| --- \| --- \| --- \| --- \| --- \| --- \| --- \| --- \| --- \| --- \| --- \| --- \| --- \| --- \| --- \| --- \| --- \| --- \| --- \| --- \| --- \| --- \| --- \| --- \| --- \| --- \| --- \| --- \| --- \| --- \| --- \| --- \| --- \| --- \| --- \| --- \| --- \| --- \| --- \| --- \| --- \| --- \| --- \| --- \| --- \| --- \| --- \| --- \| --- \| --- \| --- \| --- \| --- \| --- \| --- \| --- \| --- \| --- \| --- \| --- \| --- \| --- \| --- \| --- \| --- \| --- \| --- \| --- \| --- \| --- \| --- \| --- \| --- \| --- \| --- \| --- \| --- \| --- \| --- \| --- \| --- \| --- \| --- \| --- \| --- \| --- \| --- \| --- \| --- \| --- \| --- \| --- \| --- \| --- \| --- \| --- \| --- \| --- \| --- \| --- \| --- \| --- \| --- \| --- \| --- \| --- \| --- \| --- \| --- \| --- \| --- \| --- \| --- \| --- \| --- \| --- \| --- \| --- \| --- \| --- \| --- \| --- \| --- \| --- \| --- \| --- \| --- \| --- \| --- \| --- \| --- \| --- \| --- \| --- \| --- \| --- \| --- \| --- \| --- \| --- \| --- \| --- \| --- \| --- \| --- \| --- \| --- \| --- \| --- \| --- \| --- \| --- \| --- \| --- \| --- \| --- \| --- \| --- \| --- \| --- \| --- \| --- \| --- \| --- \| --- \| --- \| --- \| --- \| --- \| --- \| --- \| --- \| --- \| |
| --- | --- | --- | --- | --- | --- | --- | --- | --- | --- | --- | --- | --- | --- | --- | --- | --- | --- | --- | --- | --- | --- | --- | --- | --- | --- | --- | --- | --- | --- | --- | --- | --- | --- | --- | --- | --- | --- | --- | --- | --- | --- | --- | --- | --- | --- | --- | --- | --- | --- | --- | --- | --- | --- | --- | --- | --- | --- | --- | --- | --- | --- | --- | --- | --- | --- | --- | --- | --- | --- | --- | --- | --- | --- | --- | --- | --- | --- | --- | --- | --- | --- | --- | --- | --- | --- | --- | --- | --- | --- | --- | --- | --- | --- | --- | --- | --- | --- | --- | --- | --- | --- | --- | --- | --- | --- | --- | --- | --- | --- | --- | --- | --- | --- | --- | --- | --- | --- | --- | --- | --- | --- | --- | --- | --- | --- | --- | --- | --- | --- | --- | --- | --- | --- | --- | --- | --- | --- | --- | --- | --- | --- | --- | --- | --- | --- | --- | --- | --- | --- | --- | --- | --- | --- | --- | --- | --- | --- | --- | --- | --- | --- | --- | --- | --- | --- | --- | --- | --- | --- | --- | --- | --- | --- | --- | --- | --- | --- | --- | --- | --- | --- | --- | --- | --- | --- | --- | --- | --- | --- | --- | --- | --- | --- | --- | --- | --- | --- | --- | --- | --- | --- | --- | --- | --- | --- | --- | --- | --- | --- | --- | --- | --- | --- | --- | --- | --- | --- | --- | --- | --- | --- | --- | --- | --- | --- | --- | --- | --- | --- | --- | --- | --- | --- | --- | --- | --- | --- | --- | --- | --- | --- | --- | --- | --- | --- | --- | --- | --- | --- | --- | --- | --- | --- | --- | --- | --- | --- | --- | --- | --- | --- | --- | --- | --- | --- | --- | --- | --- | --- | --- | --- | --- | --- | --- | --- | --- | --- | --- | --- | --- | --- | --- | --- | --- | --- | --- | --- | --- | --- | --- | --- | --- | --- | --- | --- | --- | --- | --- | --- | --- | --- | --- | --- | --- | --- | --- | --- | --- | --- | --- | --- | --- | --- | --- | --- | --- | --- | --- | --- | --- | --- | --- | --- | --- | --- | --- | --- | --- | --- | --- | --- | --- | --- | --- | --- | --- | --- | --- | --- | --- | --- | --- | --- | --- | --- | --- | --- | --- | --- | --- | --- | --- | --- | --- | --- | --- | --- | --- | --- | --- | --- | --- | --- | --- | --- | --- | --- | --- | --- | --- | --- | --- | --- | --- | --- | --- | --- | --- | --- | --- | --- | --- | --- | --- | --- | --- | --- | --- | --- | --- | --- | --- | --- | --- | --- | --- | --- | --- | --- | --- | --- | --- | --- | --- | --- | --- | --- | --- | --- | --- | --- | --- | --- | --- | --- | --- | --- | --- | --- | --- | --- | --- | --- | --- | --- | --- | --- | --- | --- | --- | --- | --- | --- | --- | --- | --- | --- | --- | --- | --- | --- | --- | --- | --- | --- | --- | --- | --- | --- | --- | --- | --- | --- | --- | --- | --- | --- | --- | --- | --- | --- | --- | --- | --- | --- | --- | --- | --- | --- | --- | --- | --- | --- | --- | --- | --- | --- | --- | --- | --- | --- | --- | --- | --- | --- | --- | --- | --- | --- | --- | --- | --- | --- | --- | --- | --- | --- | --- | --- | --- | --- | --- | --- | --- | --- | --- | --- | --- | --- | --- | --- | --- | --- | --- | --- | --- | --- | --- | --- | --- | --- | --- | --- | --- | --- | --- | --- | --- | --- | --- | --- | --- | --- | --- | --- | --- | --- | --- | --- | --- | --- | --- | --- | --- | --- | --- | --- | --- | --- | --- | --- | --- | --- | --- | --- | --- | --- | --- | --- | --- | --- | --- | --- | --- | --- | --- | --- | --- | --- | --- | --- | --- | --- | --- | --- | --- | --- | --- | --- | --- | --- | --- | --- | --- | --- | --- | --- | --- | --- |
