# Supplementary material for: MicroRNA-181a promotes angiogenesis in colorectal cancer by targeting SRCIN1 to promote the SRC/VEGF signaling pathway
Source: Cell Death Dis. 2018 Apr 19;9(4):438. doi: 10.1038/s41419-018-0490-4 (PMC5941226; doi:10.1038/s41419-018-0490-4)
Supplement: Supplementary file 3 — Table S2 [file 41419_2018_490_MOESM3_ESM.docx]

**Supplemental Table 2.** **Meta-analysis of the miR-181a and SRCIN1 expression in patterns of 8 normal solid tissues and 457 colon adenocarcinoma**

| ID | Normal | Tumor | Fold change | P value |
| --- | --- | --- | --- | --- |
| miR-181a | 2432.99 | 33035.52 | 13.58 | <0.001 |
| SRCIN1 | 305.75 | 66.53 | 0.22 | <0.001 |
