# Supplementary material for: MicroRNA-181a promotes angiogenesis in colorectal cancer by targeting SRCIN1 to promote the SRC/VEGF signaling pathway
Source: Cell Death Dis. 2018 Apr 19;9(4):438. doi: 10.1038/s41419-018-0490-4 (PMC5941226; doi:10.1038/s41419-018-0490-4)
Supplement: Supplementary file 4 — Table S3 [file 41419_2018_490_MOESM4_ESM.docx]

**Supplemental Table 3.** **Patients’ Characteristics**

| Case number | Age | Gender | TNM stage | Cancer subtype |
| --- | --- | --- | --- | --- |
| 1 | 65 | F | III | colon carcinoma |
| 2 | 63 | M | III | colon carcinoma |
| 3 | 59 | F | III | colon carcinoma |
| 4 | 71 | F | III | colon carcinoma |
| 5 | 46 | M | IV | colon carcinoma |
| 6 | 43 | M | III | colon carcinoma |
| 7 | 60 | M | III | colon carcinoma |
| 8 | 75 | M | III | colon carcinoma |
| 9 | 50 | F | IV | colon carcinoma |
| 10 | 63 | F | III | colon carcinoma |
| 11 | 72 | M | III | colon carcinoma |
| 12 | 40 | M | IV | colon carcinoma |
| 13 | 35 | F | IV | colon carcinoma |
| 14 | 65 | M | III | colon carcinoma |
